# Elastic metamaterials for tuning circular polarization of electromagnetic waves

**DOI:** 10.1038/srep28273

**Published:** 2016-06-20

**Authors:** Yair Zárate, Sahab Babaee, Sung H. Kang, Dragomir N. Neshev, Ilya V. Shadrivov, Katia Bertoldi, David A. Powell

**Affiliations:** 1 Nonlinear Physics Centre and Centre for Ultrahigh-bandwidth Devices for Optical Systems (CUDOS), Research School of Physics and Engineering, The Australian National University, Canberra, ACT 2601 Australia; 2 John A. Paulson School of Engineering and Applied Sciences, Harvard University, Cambridge, Massachusetts 02138, USA; 3 Department of Mechanical Engineering and Hopkins Extreme Materials Institute, Johns Hopkins University, Baltimore, Maryland 21218, USA

## Abstract

Electromagnetic resonators are integrated with advanced elastic material to develop a new type of tunable metamaterial. An electromagnetic-elastic metamaterial able to switch on and off its electromagnetic chiral response is experimentally demonstrated. Such tunability is attained by harnessing the unique buckling properties of auxetic elastic materials (buckliballs) with embedded electromagnetic resonators. In these structures, simple uniaxial compression results in a complex but controlled pattern of deformation, resulting in a shift of its electromagnetic resonance, and in the structure transforming to a chiral state. The concept can be extended to the tuning of three-dimensional materials constructed from the meta-molecules, since all the components twist and deform into the same chiral configuration when compressed.

Recent advances in fabrication at micro- and macro-scales have enabled the design of metamaterials with electromagnetic properties that are distinct from their constituents, and exhibit extreme parameter values previously thought impossible^1^,^2^. Of particular interest are chiral metamaterials, which exhibit polarisation rotation and circular dichroism orders of magnitude larger than natural chiral media^3^. Chiral metamaterials have been demonstrated in microwave^4^, terahertz^5^ and near infra-red^6^ spectral ranges. Beside their ability to rotate the plane of linearly polarised waves and selectively filter by circular polarisation, their reduced symmetry makes them of great interest for the enhancement of nonlinear processes^7^.

While the first generation of electromagnetic metamaterials was characterized by properties fixed during the assembly process^8^, recently there has been considerable interest to expand their functionality by designing systems capable of changing properties reversibly through external stimuli. Techniques for achieving such dynamic tunability which are applicable to chiral media include varactor diodes^9^, liquid crystals^10^ and micro-electromechanical systems (MEMS)^11^. Reconfiguration of the internal geometry of meta-atoms has proven to be a highly effective mechanism for tuning the transmission response and controlling chirality. Notable examples of electromagnetic functionality controlled by elastic deformation include stretchable antennas using microfluidics or silver nanowires^12^,^13^. Moreover, tunable flexible metamaterials promise a new wave of device designs and functionalities^14^. In another approach, metamaterial deformation is induced using electromagnetic forces, yielding to many interesting nonlinear phenomena that range from bistability, spontaneous symmetry breaking and self-oscillations^15^,^16^. However, deformation by electromagnetic forces requires systems with an extremely soft elastic response, which often exhibit unwanted mechanical degrees of freedom. This makes them unsuitable for polarisation manipulation applications outside a controlled laboratory, such as in microwave communication, sensing or imaging, since vibrations would completely degrade performance. Therefore, an important problem in the field of tunable metamaterials is the development of an electromagnetic material with carefully designed modes of mechanical deformation.

In this communication, we propose and demonstrate the combination of advanced elastomeric metamaterials with resonant electromagnetic structures to achieve prescribed mechanical tunability of the electromagnetic response. Particularly, we exploit the unique geometric conformation that an elastic structure undergoes under compression to fabricate a composite *electromagnetic-elastic meta-molecule* with dynamic tunability. Our approach utilises an external force to deform the structure of the meta-molecule (the building block of a metamaterial), which provides precise control over its mechanical response, and therefore over its electromagnetic properties. Most importantly, the proposed mechanism of tunability is robust and can be easily scaled to three-dimensional metamaterials by using our engineered meta-molecule as a basic constituent.

Our engineered electromagnetic meta-molecule can be reversibly tuned between achiral and highly chiral states by an external force. While recently there have been a number of studies demonstrating achiral to chiral transition induced by elastic deformation in 2D structures^17^,^18^,^19^, to control circular polization of electromagnetic waves we need fully 3D structures exhibiting strong coupling with electromagnetic waves. Here this is achieved by embedding metallic elements into an engineered elastic structure, a patterned spherical shell with six circular holes known as a buckliball (see Figs 1 and 2), for which significant geometric rearrangements have been observed as a result of elastic instability^20^. This building block has previously been used as a constituent element to create tunable 3D soft negative Poisson’s ratio (auxetic) metamaterials^21^ and phononic crystals^22^. Here we exploit its unique deformation induced by buckling under uniaxial compression to design an electromagnetic-elastic meta-molecule able to reversibly tune the circular dichroism of electromagnetic waves. This is enabled by its ability to switch between achiral and chiral configurations. Since the differential absorption of left- and right-handed light is highly affected by the chirality of the medium in which the electromagnetic waves propagate^7^, it is easy to see that the design of a meta-molecule with reversibly tunable circular dichroism requires a 3D structure with variable structural chirality.

We start by investigating the effect of uniaxial compression along the diagonal direction on the configuration of the elastic structure. In particular, we focus on a spherical shell (inner diameter *d*_*i*_ = 19.8 mm and wall thickness *t* = 7.1 mm) that is patterned with a regular array of 6 slightly tapered circular voids (19.6 mm and 11.4 mm maximum and minimum diameter, respectively) made of an elastomeric material (see Fig. 1). We further conduct Finite Element (FE) simulations to explore the mechanism that leads to the structural chirality of the system (details of the FE simulations are given in Materials and Methods). In Fig. 1 we present an experimental (top) and numerical (bottom) sequence of a buckliball at different levels of applied unidirectional strain, *ε* = Δ*L*/*L*_0_. The snapshots indicate that beyond a critical strain (*ε* = −0.12), the narrow ligaments between the holes buckle on the spherical surface, leading to a cooperative buckling cascade of the skeleton of the structure. During this process each ligament rotates (in this case counterclockwise) until the holes are nearly closed at an applied strain of *ε* = −0.3. Importantly, as the structure is compressed, it twists generating enantiomorphs; each half of the sphere is deformed in a chiral form, but with the opposite handedness. Therefore, these results show that each hemisphere is a potential platform to fabricate a meta-molecule with reversible chirality.

Next, we exploit the particular geometric configuration that the structured shell achieve under compression to build a meta-molecule with an electromagnetic chiral response that can be manipulated by means of the applied load. To couple the elastic deformation with the electromagnetic response, we inserted two flexible V-shaped metallic wires into four adjacent ligaments of the buckliball (as shown in Fig. 2a). Note that, given their flexibility, the V-shaped wires do not affect the deformation of the structure and twist following the buckling shape of the rubber beams (cf. Fig. 2b). We then performed numerical simulations of the composite meta-molecule to monitor the effect of the applied deformation on the structural chirality of the meta-molecule. In particular, we considered the triangle formed by the extremes of the V-shaped wires, denoted O, A, B, M, Q and N in Fig. 2a, and then tracked them during deformation. The structural chirality index, denoted by *I*, is defined as the difference between the angles 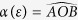 and 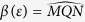 (cf. Fig. 2b).


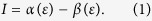


Figure 2c shows the structural chirality of the meta-molecule as function of the applied strain. For low levels of compression the proposed meta-molecule possesses a plane of symmetry and therefore it is achiral. Once the buckling threshold is exceeded (cf. blue shaded region in Fig. 2c) the meta-molecule begins to deform in a chiral fashion and the structural chirality *I* rapidly increases. Therefore, our numerical analyses reveal that in the proposed meta-molecule buckling acts as a switch between achiral and chiral configurations.

Having identified a meta-molecule whose structural chirality can be controlled by the applied deformation, we now investigate how this affects its electromagnetic response. When the sample is excited by a linearly polarized electromagnetic wave propagating in the 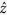 direction (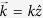), we can observe two main modes of resonance which depend on the polarization of the incident electric field relative to the orientation of the V-shaped wires. The corresponding surface current distributions are shown in Fig. 2d,e. Note that the excited modes correspond to electric (symmetric, Fig. 2d) and magnetic (antisymmetric, Fig. 2e) induced dipole moments in the absence of deformation. Compressing the structure breaks the symmetry, thus coupling these two different responses.

To characterise the tunability of the electromagnetic response of the engineered meta-molecule, we have measured the microwave transmission at different levels of compression. For this purpose, the meta-molecule is first arranged in a specially designed holder, and compressed up to the desired strain (see Supplementary information for details about the experimental set-up). The linear compression applied to the meta-molecule is always along the diametrically opposite vertices of the buckliball. Furthermore, the compression line always remains along the 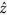 axis, independent of the stress applied to the sample (cf. Fig. 2). Consequently, the system (meta-molecule and holder) is placed into a waveguide of circular cross section, operating at microwave frequencies. We measure the scattering parameters for linearly polarized incident electromagnetic fields with wavevector along the 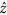 axis, 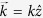, measuring both co- and cross-polarized transmission for *x* and *y* polarization of the incident wave (for details regarding electromagnetic measurements see Materials and Methods). To retrieve the electromagnetic chiral response of the meta-molecule, we transform the scattering parameters to a circular polarization basis according to the relation,


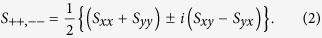


The subscripts +, − indicate circularly polarized right-handed or left-handed electromagnetic waves, respectively. The chiral response of the meta-molecule is quantified by evaluating the ratio between the right- and left-handed scattering parameters, 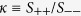. The absolute value of this quantity, abs[*κ*], gives the circular dichroism of the electromagnetic-elastic sample, while the optical activity is determined by its argument, arg[*κ*].

The electromagnetic chirality exhibited by the meta-molecule is shown in Fig. 3. The measurements were performed at different levels of compression, starting from the uncompressed state and increasing to the regime in which the meta-molecule undergoes buckling (cf. blue shaded region in Fig. 2c). It is important to mention that the maximum strain that can be applied is 30% since for higher values the ligaments of the buckliball begin to contact one another, i.e. densification starts. It can be seen that, as the applied load increases, the circular dichroism (Fig. 3a) and the optical activity (Fig. 3b) responses are enhanced and their electromagnetic resonances, highlighted by circular markers, shift to higher frequencies. Note that although ideally the meta-molecule should be achiral in the uncompressed state, it actually has a nonzero chiral response at this level of strain (cf. Fig. 3a). This is because experimentally the inserted V-shaped wires are not perfectly aligned, and their fabrication asymmetry generates this response.

Using numerical simulations, we follow a procedure similar to that of experiments. We first determine the geometrical configuration of the buckliball with V-shaped wires inclusions at different levels of applied uniaxial compression. We then perform the electromagnetic simulations for every configuration (see Numerical Simulations section in Materials and Methods). The numerical results for the circular dichroism and optical activity are shown in Fig. 3c,d, respectively. It is worth noting that in the numerical simulations a nonlinear post-buckling analysis has to be performed in order to determine the position of the wires. This process introduces imperfections to the uncompressed system which also lead to a small chiral response (cf. Fig. 3c). It can be seen that our experimental results are in very good agreement with the numerical simulations. Moreover, the frequency shift and the enhancement of the electromagnetic chiral response show nonlinear behavior.

To clarify this point, we have extracted the maximum circular dichroism as a function of the applied strain, and compared the experimental data with the numerical results in Fig. 4. It can be seen that there is a consistent disparity between the experimental and numerical data. We attribute this to the imperfections present in the manufacture of the sample (see Materials and Methods). Notwithstanding this difference, these results confirm that the chiral response of the system can be manipulated by means of compression exerted on the meta-molecule. It is noteworthy that a saturation process is observed for high values of compression (cf. Fig. 4). This occurs as a consequence of the nonlinear mechanism involved in the buckling process of the electromagnetic-elastic meta-molecule. The fundamental reason for the saturation behavior of the meta-molecule is because electromagnetic chirality is a three dimensional characteristic of materials. As the material is compressed, the V-shaped antennas become planar, which makes them less chiral (they just become anisotropic).

By comparing Fig. 2c with Fig. 4, we see a clear correlation between the structural chirality and its electromagnetic manifestation as circular dichroism. However, at higher strain levels the circular dichroism flattens off, while the structural chirality is still increasing. We attribute this to the reduced length of the entire structure with increased compression. The electromagnetic chirality requires strong artificial magnetism which in turn requires retardation of the electromagnetic wave across the structure^23^. With increasing compression both the retardation and magnetic response are reduced. Thus, the geometric chirality cannot fully characterize the electromagnetic chirality, which is to be expected since the former is a single real scalar, while the latter is a complex quantity depending on the frequency, angle of incidence and polarization of the incident wave.

Having characterized the coupled elastic and electromagnetic properties of a single meta-molecule, we now consider the collective response when meta-molecules are assembled to form a three-dimensional metamaterial. As shown in a previous study where the elastic buckliballs have been used to create 3D soft materials with negative Poisson’s ratio^21^, it is possible to create a body-centered-cubic crystal using the 6-hole pierced buckliball as a building block. Such a 3D structure is ideal for our purpose, since when compressed along two parallel faces all elements fold in the same orientation. Therefore, the chiral electromagnetic response of the 3D elastic metamaterial should be greater than that obtained from a single meta-molecule (cf. Fig. 3).

Accordingly, we numerically analyze a 3D sample created by sandwiching a layer of 5 × 5 meta-molecules with two layers of 6 × 6 meta-molecules following a BCC configuration (the unit cell of the crystal is shown in the inset of Fig. 5, different views of the metamaterial are found in the Supplementary Information). It is worth mentioning that, in the array, all the meta-molecules are joined to each other through their vertices which constrains them all to rotate in the same sense. This results in the 3D configuration each elements deforms in a different fashion to when they are compressed individually^21^. We numerically studied the electromagnetic chirality of the three-dimensional sample, when compressed along the 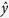 direction. For this type of compression the vector connecting the diametrically opposite vertices of the compressed buckliball changes its orientation as a function of the strain.

The sample is excited by a plane electromagnetic wave with direction of propagation along the diagonal of the uncompressed buckliball, so that the propagation direction has a consistent relationship with the axes of the sample. This differs from what was done with the meta-molecule where the compression line and the direction of propagation of electromagnetic wave were always parallel, independent of the compression. Since the structure is finite-sized and may scatter in arbitrary directions, the circular dichroism and optical activity are calculated for the scattered wave in the forward direction, rather than the transmitted wave used for the single structure. These quantities are plotted in Fig. 5. The structure features a qualitatively similar electromagnetic response to that obtained from the single meta-molecule, despite the differences in the excitation scheme. This shows that the wave manipulation principle experimentally demonstrated for a single element is equally applicable to the manipulation of a beam in free space incident upon a bulk sample. We expect that this structure should also be able to exhibit more complex manipulation of near and far-fields if subject to an inhomogeneous force distribution.

In summary, an engineered meta-molecule with a tunable chiral electromagnetic response has been studied by coupling elastic deformation with electromagnetic phenomena. The circular dichroism of electromagnetic waves can be controlled by means of the strain exerted on the system. The electromagnetic chirality of the meta-molecule was measured as function of the strain and the results showed good agreement with numerical simulation. Interestingly, the meta-molecule exhibits nonlinear mechanical and electromagnetic behavior upon uniaxial compression, generating a chiral structure with circular dichroism. The geometric and electromagnetic chirality are correlated for low levels of compression but as the strain increases they depart from each other. The tunability shown here for a single meta-molecule can be easily extended to three-dimensional structures since the elastic buckliballs can be stacked to create three-dimensional structures such as soft auxetic materials. We have demonstrated this numerically by designing a 3D elastic metamaterial using our experimentally verified meta-molecules as building blocks. Our numerical study showed that the three-dimensional sample exhibits the tunability of its electromagnetic chirality of as the single meta-molecule does, but with a stronger response.

Application of this concept to other forms of engineered elastic materials would enable meta-molecules allowing the manipulation of other electromagnetic properties. In particular, we expected that tunable metasurfaces could be controlled through simple unidirectional deformation. We envision that our approach can open new ways of controlling electromagnetic wave propagation by coupling three dimensional elastic deformation with the electromagnetic response. For example, they may find application in “smart transformation optics”, whereby a functionality such as cloaking or waveguiding can be maintained despite strong deformation of the structure’s geometry^24^.

## Methods

### Materials

A silicone-based rubber (commercial name: Elite Double 32, Zhermack) was used to cast the experimental specimen. The material properties were measured through tensile testing, up to a true strain of *ε* = 0.60. No hysteresis was found during loading and unloading. The measured response is accurately captured by a Yeoh hyperelastic model^25^, whose strain energy is 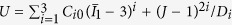 where *C*_10_ = 131 KPa, *C*_20_ = 0 KPa, *C*_30_ = 3.5 KPa, 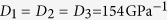. Here, 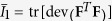, *J* = det**F**, and **F** is the deformation gradient. Two of the Yeoh model parameters are related to the conventional shear modulus, denoted by *G*_0_, and bulk modulus, denoted by *K*_0_, at zero strain: *C*_10_ = *G*_0_/2, *D*_1_ = 2/*K*_0_.

### Fabrication of the meta-molecule

A mold was fabricated using a 3-D printer (Objet Connex500) to cast one half of a spherical shell. After de-molding, two halves were joined using the same polymer as adhesive agent. The specimen fabricated for this study has the thickness of t = 7.1 mm, the inner diameter of *d*_*i*_ = 19.8 mm, and the outer diameter of *d*_*o*_ = 34.0 mm. Then, two flexible V-shaped antennas are inserted at 3 mm depth from the rubber surface. Several knots of synthetic cord are used to maintain the wires inside the rubber during the load. The external length of the meta-atom is 25.4 mm whereas the total length of the V-shaped wires is 30 mm, making the composite metamaterial suitable to work at microwave frequencies.

### Experimental Measurements

To measure the electromagnetic chirality as a function of the compression, we fabricated a frame with movable walls of styrofoam which is essentially invisible to microwave radiation. The rubber behaves as a dielectric at these frequencies with a permittivity of *ε*_*r*_ = 2.5 and loss tangent of tan(*δ*_*r*_) = 0.01. These values were determined by adjusting the numerical results for the scattering parameters for linear polarization of the uncompressed sample with those obtained experimentally. The measurements are done using a vector network analyzer Rohde & Schwarz ZVB 20.

### Numerical Simulations

**Figure 5:**
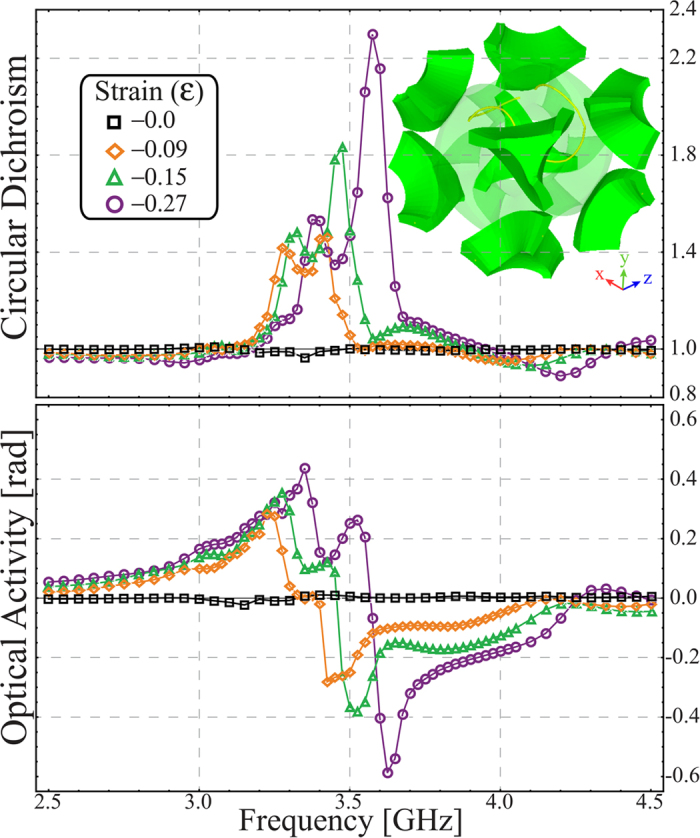
Circular dichroism (top) and optical activity (bottom) of the 3D auxtic metamaterial for different levels of strain obtained from numerical simulations. The inset shows the unit cell, a central meta-molecule surrounded by eighths of meta-molecules on each of its corners, of the three-dimensional metamaterial (BCC crystal) for the strain of *ε* = −0.27.

1. Single meta-molecule: The mechanical simulations were carried out using the commercial Finite Element package Abaqus (SIMULIA, Providence, RI). The Abaqus/Standard solver was employed for instability and post-buckling analyses. The 3D Model was built using quadratic solid elements (Abaqus element type C3D10M) with a mesh seed size of 1 mm, along with linear beam elements (Abaqus element type B31) with a mesh size of 0.25 mm embedded inside the model to account for the V-shaped antennas. The accuracy of each mesh was ascertained through a mesh refinement study. The analyses were performed under uniaxial compression. We used first four eigenvalues from instability analysis as imperfection on nonlinear post-buckling analysis. To obtain the electromagnetic linear scattering parameters, we used the frequency domain solver of the commercial software CST Microwave Studio. Then we calculate the numerical electromagnetic chirality by evaluating Eq. (2).
2. 3D metamaterial: In order to obtain the electromagnetic chirality of the 3D auxetic metamaterial, we simulate a 3D sample created by sandwiching a layer of 5 × 5 meta-molecules with two layers of 6 × 6 meta-molecules following a BCC configuration whose unit cell (formed by a central meta-molecule, surrounded by eighths of meta-molecule on each of its corners), at the strain of *ε* = −0.27, is shown in the inset of Fig. 5. Due to the high computational burden of the complex structure, we replace the elastic material by an effective homogeneous background material. This approach is justified since the chiral response is dominated by the metal wires. Comparison simulations on a small finite sample confirms that this process under-estimates the chirality of the scattered wave, so our reported results give a lower bound on the performance of the structure. For each level of compression (cf. Fig. 5) we excite the system with a electromagnetic plane wave, and recorded the scattered far-field using a probe along the direction of propagation. We then retrieve the chiral parameter by transforming the forward scattered field from a linear to circular basis as per Eq. (2).

## Additional Information

**How to cite this article**: Zárate, Y. *et al.* Elastic metamaterials for tuning circular polarization of electromagnetic waves. *Sci. Rep.* **6**, 28273; doi: 10.1038/srep28273 (2016).

## Supplementary Material

Supplementary Information

## Figures and Tables

**Figure 1:**
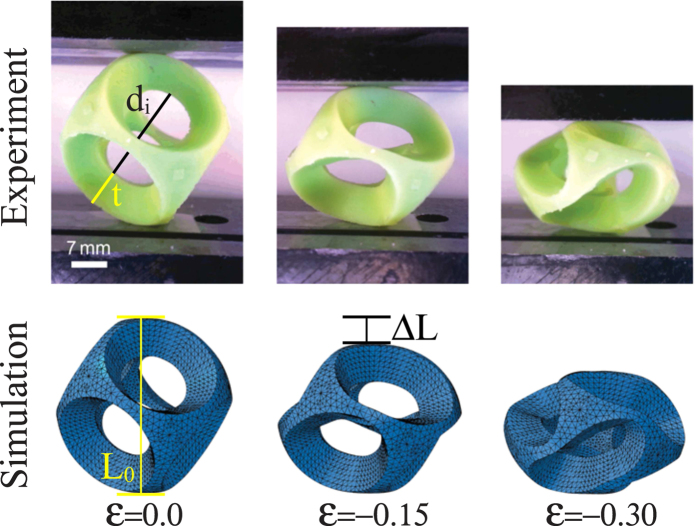
Experimental and numerical images of the buckliball, with inner diameter *d*_*i*_ = 19.8 mm and wall thickness *t* = 7.1 mm, at different levels of applied strains, *ε* = Δ*L/L*_0_.

**Figure 2:**
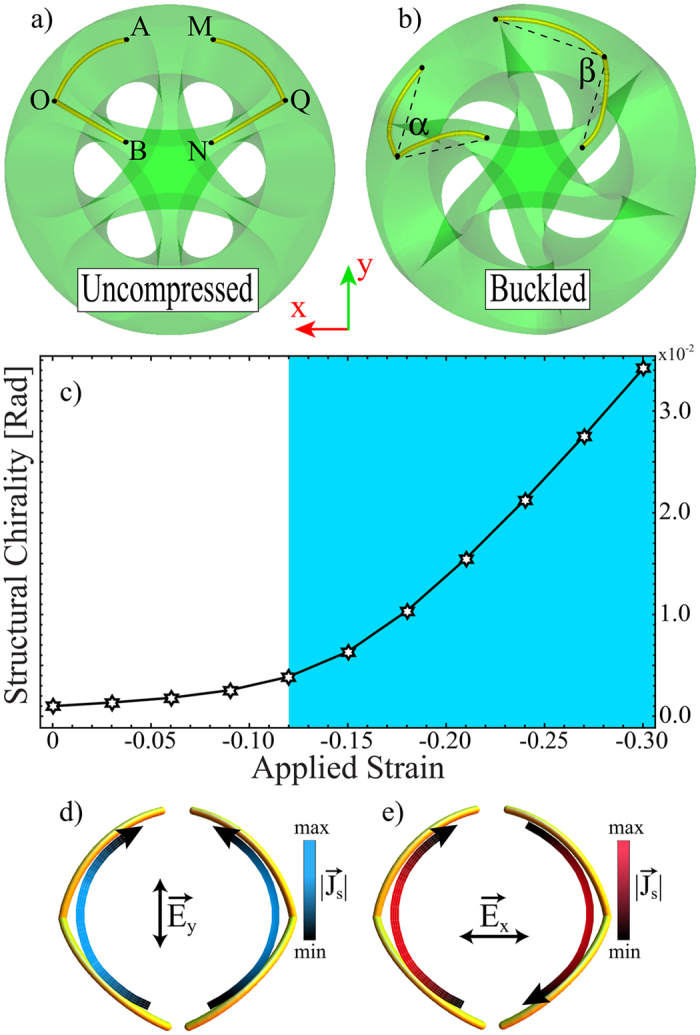
Engineered meta-molecule made of a buckliball (a rubber cube with six holes) with metallic insertions. (**a**) The sample is symmetric in the uncompressed state (**b**) however, it becomes chiral when compressed. (**c**) Evolution of the structural chirality index, measured in radians, as a function of applied strain. The blue shaded region represents the strain at which buckling occurs (i.e. −0.30 ≤ *ε* ≤ −0.12). Shown in (**d**,**e**) are the two resonance modes and the resulting surface current distribution after exciting the sample with a linearly polarized electromagnetic wave. They correspond to (**d**) symmetric, and (**e**) antisymmetric modes.

**Figure 3:**
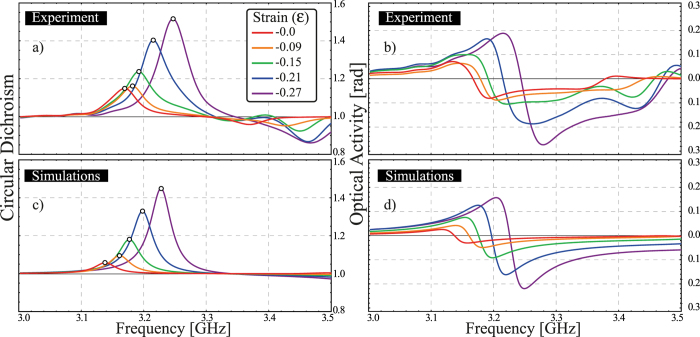
Electromagnetic chirality of the engineered meta-molecule. Experimental (**a**) and numerical (**c**) circular dichroism at various strain values. (**b**,**d**) show the achieved optical activity (measured in radians) for the experiment and the simulations, correspondingly. Both quantities have been retrieved from the linear scattering parameter through Eq. (2).

**Figure 4:**
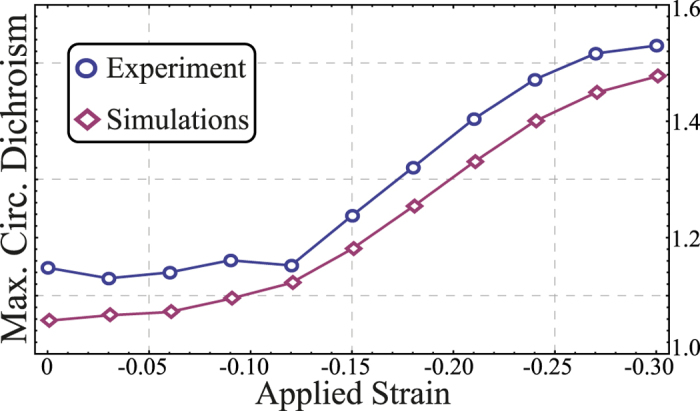
Comparison of the experimental (⚬) and numerical (◊) maximum circular dichroism. It can be seen that for low levels of compression the electromagnetic behavior (qualitatively) matches with the structural buckling (cf. Fig. 2c), however they differ for high levels of compression where the V-shaped antennas become flat.
