# Supplementary material for: Elastic metamaterials for tuning circular polarization of electromagnetic waves
Source: Sci Rep. 2016 Jun 20;6:28273. doi: 10.1038/srep28273 (PMC4913306; doi:10.1038/srep28273)
Supplement: Supplementary Information [file srep28273-s1.pdf]

Supplementary Material

Elastic Metamaterials for Tuning Circular Polarization of Electromagnetic Waves

Yair Zarate[1], Sahab Babaee[2], Sung H. Kang[2,3], Dragomir N. Neshev[1], Ilya V. Shadrivov[1], Katia Bertoldi[2], David A. Powell[1]

[1]Nonlinear Physics Centre and Centre for Ultrahigh-bandwidth Devices for Optical Systems (CUDOS), Research School of Physics and Engineering, The Australian National University, Canberra, ACT 2601, Australia

[2]John A. Paulson School of Engineering and Applied Science, Harvard University, Cambridge, MA 02138, USA

[3]Department of Mechanical Engineering and Hopkins Extreme Materials Institute, Johns Hopkins University, Baltimore, Maryland 21218, USA

S1 Experimental Details

Our meta-molecule is built by inserting two flexible V-shaped wires into a pierced spherical shell with 6 holes (a buckliball) [1]. Figure S1a shows the designed meta-molecule, which dimensions are: external length L=25.4 mm, beam thickness s=2.54 mm, radial thickness t=7.1 mm, and inner diameter of di=19.8 mm.

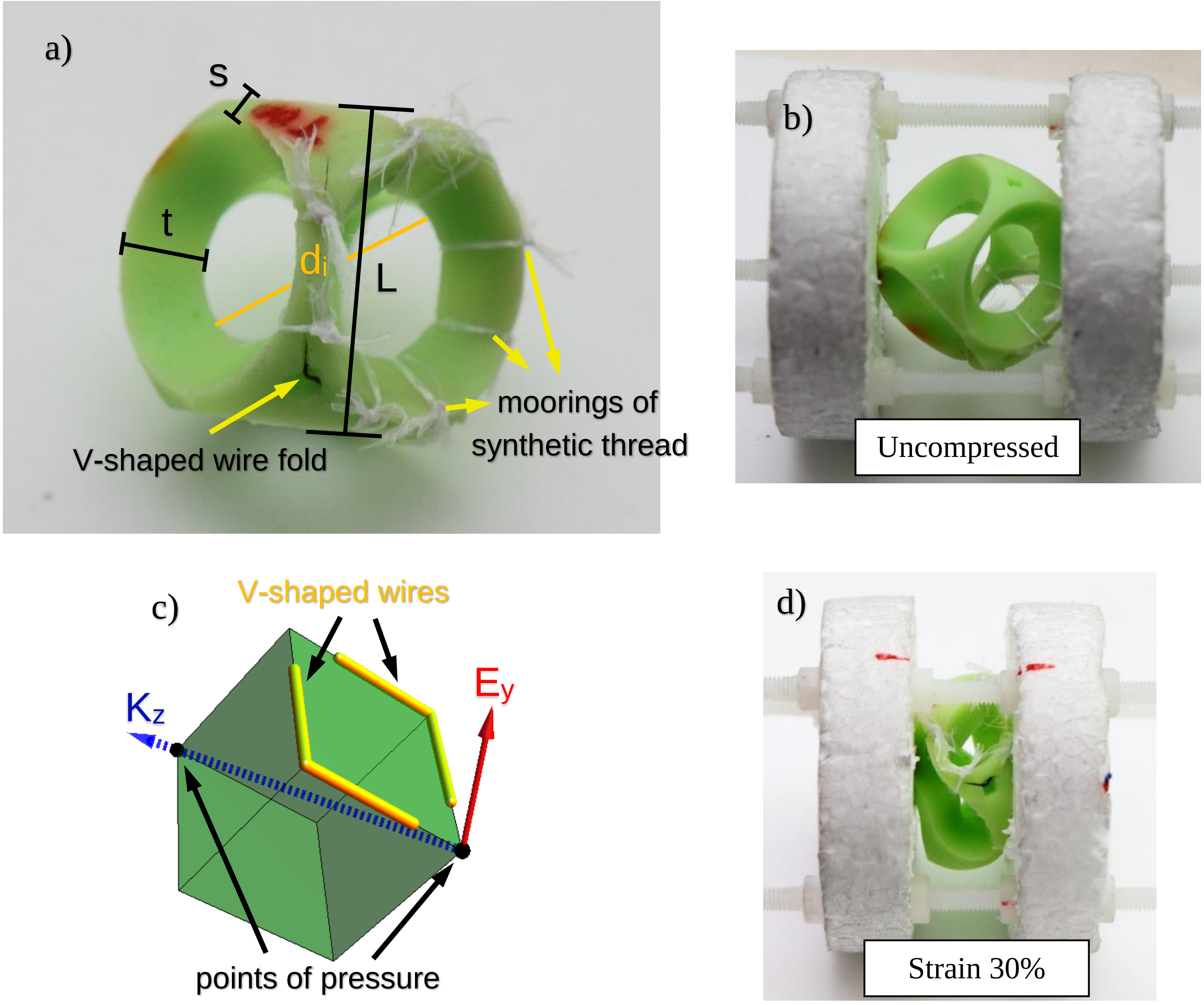

Figure S1: a) The proposed meta-molecule is built by inserting two flexible V-shaped wires in the chosen buckliball [1]. b) In order to measure the electromagnetic properties of the electro-elastic metamaterial at different level of compression, the sample is placed in a specially designed holder with movable walls of Styrofoam. c) The relative orientation of the V-shaped wires to the incident linear polarized electromagnetic field, the blue dashed line accounts for the direction of propagation of the EM field ($\vec{k} = k_z\,\hat{z}$) and it coincides with the compression line. d) Shows the meta-molecule compressed to 30% before being inserted in the waveguide of circular cross section of the same radius.

As shown in Figure S1, the V-shaped wires (copper) have been placed inside the rubber cube. To achieve this, we made straight cuts along the beams of the cube. The wires are in opposite vertices of the same side of the cube. The wire fold matches with the cube vertex. Once the wires were arranged, we tied several windings of synthetic thread to hold them inside the auxetic cube. The dimensions of the V-shaped (copper) wires are: total length 30 mm (two arms of 15 mm each), diameter 0.35 mm. The wires are at a depth of 3 mm into the rubber beam (measured from the outer surface).

To measure the optical chiral response of the auxetic metamaterial we fabricated a movable holder with cylindrical walls (Styrofoam) of radius 29.5 mm guided by plastic screws (cf. Figure S1b). As has been pointed out in the previous figure, the contact points (which determine the direction of compression) correspond to diametrically opposite vertices of the buckliball (cf. Figure S1c). The system (sample plus holder) is then placed in a cylindrical waveguide, with same radius of the Styrofoam holder, working at microwave frequencies. The sample is illuminated with a linear polarized electromagnetic field with direction of propagation along the $\hat{z}$ axis ($\vec{k} = k_z \hat{z}$) that always coincides with the compression line (see Figure S1c). For every electromagnetic measurement, the sample is first compressed (as an example see Figure S1d) and then placed inside the waveguide.

S2 Simulations 3D Auxetic Metamaterial

Figure S2 shows the 3D auxetic metamaterial created by sandwiching a layer of 5x5 meta-molecules with two layers of 6x6 meta-molecules following a body-centered cubic crystal configuration. Notice that the elastic buckliballs have previously been used to create 3D soft materials with negative Poisson's ratio. Such a 3D structure is ideal for our purpose, since when compressed along two parallel faces all elements fold in the same orientation. Therefore, the chiral electromagnetic response of the 3D elastic metamaterial should be greater than that obtained from a single meta-molecule.

We numerically studied the electromagnetic chirality of the three-dimensional sample, when compressed along the $\hat{y}$ direction. For this type of compression the vector connecting the diametrically opposite vertices of the compressed buckliball changes its orientation as a function of the strain. The sample is excited by a plane electromagnetic wave with direction of propagation along the diagonal of the uncompressed buckliball, i.e. $\vec{k} = (1, -1, 1)/\sqrt{3}$, so that the propagation direction has a consistent relationship with the axes of the sample. This differs from what was done with the meta-molecule where the compression line and the direction of propagation of electromagnetic wave were always parallel, independent of the compression.

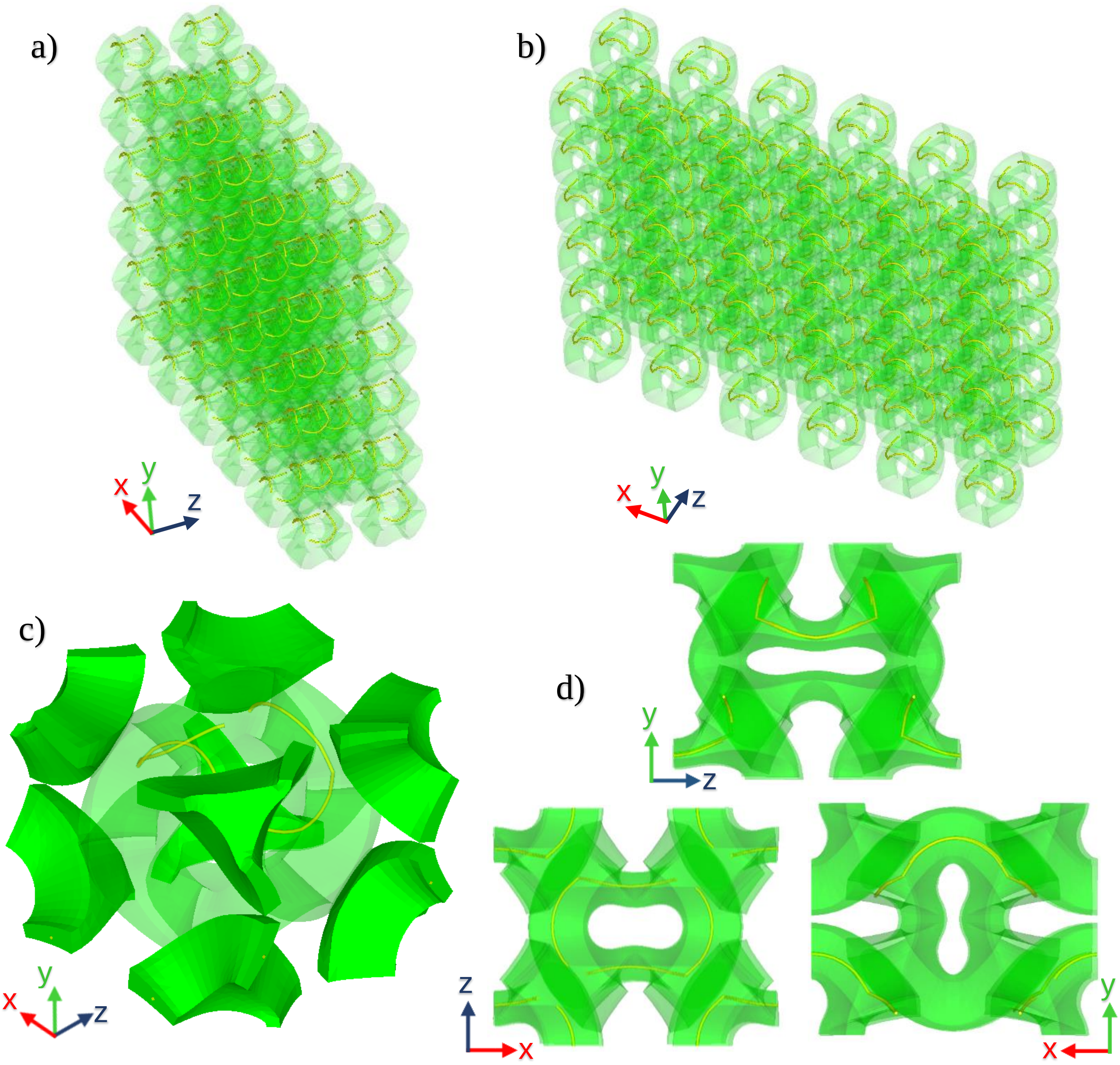

Figure S2: a) and b) Views of the proposed 3D chiral metamaterial built by sandwiching a layer of 5x5 meta-molecules with two layers of 6x6 meta-molecules following a bodycentered-cubic crystal configuration at the strain of $\epsilon = -0.27$. c) Unit cell of the BCC EM chiral crystal, it is formed by a central meta-molecule surrounded by eighths of meta-molecule on each of its corners. In d) are shown different views of the unit cell where it can be seen the pieces of wires inside the eights of meta-molecule.

References

[1] S. Babaee, J. Shim, J. C. Weaver, E. R. Chen, N. Patel, K. Bertoldi, Adv. Mater. 2013, 25, 5044.
